# Factors Related to Job Continuance of Nurses Who Migrated to Japan: A Cross-Sectional Study

**DOI:** 10.3390/nursrep14010003

**Published:** 2023-12-23

**Authors:** Rina Shoki, Anna Kono, Yuko O. Hirano, Edward Barroga, Erika Ota, Yasuko Nagamatsu

**Affiliations:** 1 Graduate School of Nursing Science, St. Luke’s International University, 10-1 Akashi-cho, Chuo-ku, Tokyo 104-0044, Japan; ota@slcn.ac.jp (E.O.); sarah-nagamatsu@slcn.ac.jp (Y.N.); 2 Department of Nursing, Faculty of Health Care, Teikyo Heisei University, 4-21-2 Nakano, Nakano-ku, Tokyo 164-8530, Japan; a.kono@thu.ac.jp; 3 Institute of Biomedical Science, Nagasaki University, 1-7-1 Sakamoto, Nagasaki-shi, Nagasaki 852-8520, Japan; hirano@nagasaki-u.ac.jp; 4 Department of Medical Education, School of Medicine, Showa University, 1-5-8, Hatanodai, Shinagawa-ku, Tokyo 142-8555, Japan; edward-barroga@slcn.ac.jp; 5 Tokyo Foundation for Policy Research, 34F Roppongi Grandtower, 3-2-1 Roppongi, Minato-ku, Tokyo 106-6234, Japan

**Keywords:** Economic Partnership Agreement, foreign nurse, international nurse, interview, job continuance, nurse migration, qualitative study, questionnaire

## Abstract

Japan has accepted nurses from Indonesia, the Philippines, and Vietnam under the Economic Partnership Agreement, but nearly half of them have already left the workforce. This study aimed to clarify the factors related to the job continuance of nurses who migrated to Japan under the Economic Partnership Agreement. Our goal was to explore factors specific to migrant nurses and to contribute to the development of support measures for them. This research was a cross-sectional study in which a web-based questionnaire and interview were conducted at a single point in time. Migrant nurses (*n* = 40) participated in the web-based questionnaire survey. Of those, nine nurses were also interviewed. Spearman’s rank correlation coefficient was used to establish correlations, and qualitative descriptive analysis was used to analyse interviews. The questionnaire survey results revealed the content of work, human relationships in the workplace, the number of night shifts, and satisfaction with the balance between work and private life were significantly and negatively correlated with the Intention to Quit scale total score. All correlation coefficients were less than 0.5, indicating weak correlations. Three categories emerged from the interviews as positive factors related to job continuance: “generous support from the workplace”, “beneficial nursing experience in Japan”, and “determination to live in Japan”. A good working environment, generous support from the supervisor and colleagues, high values of nursing in Japan, and desire to migrate to Japan were the factors that had a positive effect on migrant nurses’ job continuance in Japan. Understanding the characteristics of migrant nurses and providing generous support will enable them to continue working in Japan. This study was not registered.

## 1. Introduction

The World Health Organization (WHO) reports that nurses and midwives constitute more than 50% of the global health workforce shortage [1]. Consequently, many developed countries invite migrant nurses to fill their nursing shortage [2]. In particular, nurses from developing countries migrate to developed countries, mainly western countries, for better salaries and working conditions, professional development, and improved living standards [2,3]. Approximately 3.7 million nurses worldwide are working outside their country of birth or training as nurses [4]. Previously, developed countries such as the US, the UK, Australia, and Saudi Arabia were the main host countries for Asian nurses from the Philippines, India, China, and Vietnam [2,5,6]. However, in recent years, Asian countries have faced a serious shortage of nurses owing to their ageing societies, and hosting migrant nurses is attracting attention as an immediate solution [7]. In particular, Japan has become the new destination for migrant nurses, as the country has addressed the shortage by introducing policies to facilitate the inflow of the skilled labour [7].

Japan has accepted nurses from Indonesia in 2008, the Philippines in 2009, and Vietnam in 2014, based on the Economic Partnership Agreement (EPA) [8]. Under this agreement, migrant nurses who have obtained a nursing qualification in their home country and have several years of work experience have been accepted to work as nursing assistants in Japanese medical facilities on the condition that they obtain a Japanese nursing license [8]. Migrant nurses who passed the Japanese national nursing examination and obtained a Japanese nursing license were allowed to work in Japan without any restrictions on the length of their employment [8]. According to a report by the Ministry of Health, Labour and Welfare [9], 413 (29%) of the 1421 nurses who came to Japan under the EPA between 2008 and 2019 obtained Japanese national nursing qualifications. However, of those who had passed the Japanese national nursing examinations, 67% in Indonesia, 42% in the Philippines, and 58% in Vietnam had already left Japan [10]. This is quite high considering that the turnover rate for Japanese nurses was 11.6% [11]. Support measures to enable migrant nurses who have passed the national nursing examination to continue working in Japan are an important issue for migrant nurses’ careers and for Japan, which faces a serious shortage of nursing personnel in the future.

For migrant nurses who came to Japan under the EPA, it is not easy to pass the Japanese national nursing examination, with only 11.9% passing the exam in 2022 [12]. Lack of Japanese language proficiency is a major obstacle to passing the national examination [13]. However, even after passing the initial hurdles of becoming qualified to work as a nurse, migrant nurses have experienced difficulties with the Japanese language when speaking in technical terms [14], attending conferences, making phone calls, and keeping nursing records [15]. In addition, differences in nursing practices and working processes between Japan and their home country [14], heavier responsibilities than when they were nurse candidates [16], and fewer opportunities to perform nursing practice independently than newly graduated Japanese nurses [17] were reported as difficulties in working in Japan.

The reasons for migrant nurses’ turnover were career development in their home country, and marriage and family care [10]. However, the various difficulties experienced in Japan [14,16,17] are also considered relevant to their turnover. One factor that generally affects nurses’ turnover is burnout [18,19]. Burnout is defined as a syndrome of emotional exhaustion, depersonalisation with unfeeling, and reduced personal accomplishment [20]. Factors affecting nurse burnout include high working hours [18,19], stressful work environment [19], inadequate staffing [19], and increased exposure to patient deaths [18]. Migrant nurses who are unfamiliar with the new language are more likely to experience burnout [21]. On the other hand, migrant nurses come to Japan for their own career development and to provide financial support to their families [22]. It has been reported that migrant nurses find higher salaries than in their home countries and a safe life in Japan meaningful [14].

Although previous studies have clarified migrant nurses’ purposes for working in Japan and reasons for leaving, as well as difficulties and burnout that may lead to turnover, there are few reports on factors related to job continuance. Studies of Japanese nurses found that higher salary [23,24], number of holidays and night shifts [25,26], good relationships with colleagues [24,26], nursing expertise [23,24], and training opportunities [26] were related to job continuance. A case study of two Indonesian nurses reported that the improvement of their Japanese language and nursing practice skills, building relationships with their colleagues, and acquiring an identity as a Japanese nurse supported them in working at a Japanese hospital [27].

However, no study has yet focused on migrant nurses who have passed the national nursing examination and identified factors related to job continuance, regardless of nationality or facility of employment. Clarifying factors related to the job continuance of migrant nurses may contribute to the development of support measures for them to continue working.

### Study Purpose

This study aimed to clarify factors related to the job continuance of migrant nurses who came to Japan under the EPA and passed the national nursing examination. We investigated whether factors related to job continuance among Japanese nurses were similar to those of migrant nurses, and explored factors specific to migrant nurses.

## 2. Materials and Methods

### 2.1. Study Design

This research used both a quantitative cross-sectional study design to explore correlation associations based on a web questionnaire survey and a qualitative descriptive design [28] with interviews. The web questionnaire provided an overview of factors related to job continuance among migrant nurses based on studies conducted with Japanese nurses. Interviews were conducted to understand factors specific to migrant nurses that may not be evident in the questionnaire. Both data collection methods were deemed appropriate to provide invaluable descriptions where none had been found in the literature. 

### 2.2. Definition of Term

“Job continuance” in this study was defined as continuing to work as a nurse in Japan. It was not limited to the workplace at the time of the survey but also included changing jobs as a nurse in Japan.

“Economic Partnership Agreement” is a treaty that promotes cooperation and collaboration in a wide range of areas and aims to strengthen bilateral or multilateral relationships [8].

### 2.3. Participants

Eligible participants were migrant nurses who came to Japan under the EPA and worked as nurses in Japan after passing the Japanese national nursing examination. The hospitals where the migrant nurses were working in Japan were not disclosed to the public unless they gave permission to the Ministry of Health, Labour and Welfare (MHLW). Thereafter, the MHLW annually disclosed the hospitals where the migrant nurses who had passed the national nursing examinations worked and the number of migrant nurses who had passed the examination. Thus, in the present study, the sample frame became the 137 hospitals which were disclosed as work facilities for 361 nurses who passed the national nursing examination from 2009 to 2019. These hospitals were asked to introduce eligible study participants to the web-based questionnaire survey. We did not set any criteria for hospitals, because we wanted to collect data from as many participants as possible. Additionally, the last question of the web questionnaire asked whether the participant would be willing to participate in an interview. Those who responded that they could participate in the interview were recruited as interview participants.

### 2.4. Data Collection

#### 2.4.1. Web-Based Questionnaire Survey

The documents for the research explanation and web questionnaire were prepared in 4 languages (i.e., English, Indonesian, Vietnamese, and Japanese) so that participants could choose the language they could easily comprehend. The English, Indonesian, and Vietnamese documents were translated by nursing researchers or translation companies whose first language are those languages. The lead researcher (RS) then performed back-translation and asked the translator to correct any differences from the researcher’s intended content.

- Basic attributes

Data were collected on the following basic attributes: age, gender identification, country of origin, marital and child status, number of visits to Japan as a nurse, length of stay in Japan, years of experience as a nurse in Japan, and years of experience as a nurse in their own country.

- Intention to Quit (3 items)

As there was no scale to measure the desire of migrant nurses to leave their jobs, the Intention to Quit scale (Cronbach’s alpha coefficient 0.645) [29], which correlated with other similar scales and whose reliability has been confirmed, was partly adopted after obtaining permission from the developer. This scale was developed based on the process of nurses’ turnover intention: ‘I want to change my current department’, ‘I want to change my current hospital’, and ‘I want to quit my job as a nurse’.

As this study was conducted with migrant nurses, the third item of the scale was reformatted to ‘I want to quit my job as a nurse in Japan’. Each item of the Intention to Quit scale was scored as 5 points for ‘always’, 4 points for ‘sometimes’, 3 points for ‘neither’, 2 points for ‘not very much’, and 1 point for ‘never’, and then calculated as a total score with a higher score indicating a stronger desire to quit their job. Total scores range from 3 to 15.

- Current Job Satisfaction (7 items)

A questionnaire about satisfaction with their current job was developed by the lead researcher (RS) from previous studies on job continuity and turnover among Japanese nurses [23,24,25,26] and categories related to meaningful experiences of migrant nurses in Japan who came to the country under the EPA [14]. Data were collected on 7 items: ‘salary’, ‘content of work’, ‘support from their supervisor’, ‘human relationships in workplace’, ‘training in workplace’, ‘number of night shifts’, and ‘balance between work and private life’. Each item was scored as 5 points for very satisfied to 1 point for not satisfied, with a higher score indicating greater job satisfaction. Total scores range from 7 to 35.

#### 2.4.2. Interview

Semi-structured online interviews were conducted for approximately 60 min using the following interview guide: (1) Please tell me about your current work. (2) Have you ever wanted to quit your job? Please tell me what kind of situation it was. (3) Why have you been able to continue your work despite the difficulties you have faced? (4) What kind of support helped you to continue working? (5) What makes you want to continue working in Japan?

Interviews were conducted on a one-to-one basis in a private environment. The content was recorded on an IC recorder with the participant’s consent. It was explained in advance that an interpreter could be provided during the interview. As there was no request for an interpreter from the participants, the interviews were conducted in Japanese. Since the interviews were conducted in a non-native language of the participants, the interviewer spoke slowly in easy-to-understand Japanese and checked the participant’s understanding of the questions.

### 2.5. Data Collection Period

This study was conducted from July 2020 to March 2021. The study participants were recruited from July to August 2020, and interviews were conducted from August to October 2020.

### 2.6. Data Analysis

Data triangulation was conducted with a quantified model of factors related to job continuance collected by a web-based questionnaire survey and qualitative data on job continuance based on individual experiences collected by interviews. 

#### 2.6.1. Web-Based Questionnaire Survey

For each of the basic attributes, a basic statistic was calculated. Confirming that the total intention to quit scores were not a normal distribution, medians were compared using the Mann–Whitney U-test (gender identification, marital status, presence of children, and whether or not they had visited Japan) and the Kruskal–Wallis’ test (country of origin). In addition, the correlation between the total score of intention to quit and age, length of stay in Japan, years of experience as a nurse in Japan, years of experience as a nurse in their own country, and satisfaction with their current job was analysed using Spearman’s rank correlation coefficient. SPSS Ver. 24.0 (IBM) was used for data analysis with a *p*-value of <0.05 indicating a statistically significant difference. 

#### 2.6.2. Interview

The qualitative descriptive research method of Gregg et al. [30] was used to analyse the interview data as follows. After transcribing all interview content verbatim, the researcher read the verbatim several times and then summarized the content relevant to job continuance using the participant’s own words. Each summary was coded by content and the code names were modified comparing similarities and differences in the content. Similarities were categorized based on the code names to identify the three major categories of factors related to job continuance. Referring to Greg et al.’s [30] Rigor Review of Analytical Results, the content of the verbatim transcripts and the results of the analysis were checked by the participants, and the process of clarifying code names and categories was supervised by a co-researcher with extensive experience in qualitative research. The validity of the results of the analysis was ensured through repeated discussions on data coding and code names with 2 study members: a graduate student and a researcher with extensive experience in qualitative research.

### 2.7. Ethical Consideration

The Research Ethics Review Committee of St. Luke’s International University approved the study (approval number: 20-A027). The participants were given a written explanation about the purpose of the study, methods, voluntary nature of participation and freedom to discontinue with the study, anonymity, privacy, publication of data, and the advantages and disadvantages of participating in this study. Data were stored with a password, and study results were published in a form that did not identify individuals.

## 3. Results

### 3.1. Web-Based Questionnaire Survey 

A total of 361 study participation request forms were sent to 137 hospitals, and 45 responses were received. Of these, 40 responses were included in the analysis, excluding duplicate respondents and responses from those who did not meet the eligibility criteria for study participants. Of these 40 responses, 10 responses for length of stay in Japan and 1 response for satisfaction with the number of night shifts were missing values. 

#### 3.1.1. Characteristics of the Participants in the Web-Based Questionnaire Survey 

The average age of the participants was 32.3 ± 4.2 years, with 11 men and 29 women. Sixteen were from Indonesia, 14 from the Philippines, and 10 from Vietnam; 53% were married and 43% had children. The majority (90%) of the respondents came to Japan for the first time as nurses. The length of stay in Japan ranged from 1 to 12 years, with an average of 5.6 ± 3.6 years. The average number of years of experience as a nurse in Japan was 3.9 ± 3.1 years, ranging from 7 months to 10 years, whereas the average number of years of experience as a nurse in their own country was 3.7 ± 1.6 years (Table 1).

#### 3.1.2. Relationship between Total Intention to Quit Scores and Basic Attributes and Satisfaction with Current Job 

The mean of the Total Intention to Quit scores was 7.9 ± 3.3 (range 3–12). A comparison of median values and correlation analysis between Total Intention to Quit scores and basic attributes showed that the items were not significantly different. However, the relationships between Total Intention to Quit scores and Satisfaction With Current Job, ‘content of work’, ‘human relationships in workplace’, ‘number of night shifts’, and ‘balance between work and private life’ were significant. The higher the satisfaction scores on ‘content of work (r = −0.453)’, ‘human relationships in workplace (r = −0.353)’, ‘number of night shifts (r = −0.394)’, and ‘balance between work and private life (r = −0.437)’, the lower the total score of Intention to Quit (Table 2).

### 3.2. Interview

#### 3.2.1. The Interview Participants

The researcher requested participants interested in the interviews to provide their email addresses at the end of the questionnaire, and successfully contacted nine participants. The researcher then arranged interview dates with the participants via email.

The nine interview participants consisted of three Indonesians, three Filipinos, and three Vietnamese. Of these nine participants, three were in their twenties and six were in their thirties; four were men and five were women; six were married and three had children. Each participant was separately interviewed for 40–60 min (Table 3). In consideration of participant privacy, interviews were audio recorded only.

#### 3.2.2. Results of Interview Analysis 

From the interview data analysis, three major factors related to job continuance of migrant nurses were extracted: Generous Support from the Workplace, Beneficial Nursing Experience in Japan, and Determination to Live in Japan. Additionally, eight categories were extracted: “dependable supervisor”, “good relationships with colleagues”, “comfortable working environment”, “high values of Japanese nursing practice”, “goals to achieve in Japan”, “attachment to current job”, “attractive life in Japan”, and “migrated to Japan with family”. Also, 31 subcategories were identified (Table 4).

- Generous Support from the Workplace

Migrant nurses were able to continue their work because of the generous support they received from their workplaces. This major factor consists of three categories: “dependable supervisor”, “good relationships with colleagues”, and “comfortable working environment”. Subcategories and narrative examples are added to explicate the categories.

1. Dependable supervisor

For this category, the participants, nursing administrators, and head nurses in their workplace showed the following characteristics: ‘easy to ask them for help’, they ‘trusted and entrusted migrant nurses’ work’, and ‘applauded their work’ by encouraging the Japanese staff to support them. Supervisors were generous in supporting migrant nurses not only in their work but also in their daily lives by assisting them in ‘resolving difficulties in work and private life’ and ‘enabling them to achieve their preferred working style’ in terms of assignments and holidays.

“The (nursing) administrator is very kind and easy to ask [for assistance].” (More than 5 years.)

“My superiors didn’t think that because I’m a foreigner and my Japanese is difficult, I might not be able to be a leader. I am glad to that they trust and accepted that because I am a nurse, I am the same as Japanese.” (More than 5 years.)

“One year after I passed the Japanese national nursing examination, my family came to Japan, and the nursing administrator was really supportive then. [My spouse has] no experience [in caring], but [the hospital] gave [my spouse] a job as a care worker.” (More than 5 years.)

“When I said, ‘I want a longer holiday’, [My supervisor asked me] ‘When?’, ‘How long do you want?’. They have never said no.” (More than 5 years.)

2. Good relationships with colleagues

For this second category, the Japanese colleagues ‘understand the capability of migrant nurses’ and ‘kindly teach them’, and ‘support migrant nurses with unfamiliar tasks’ and ‘help them with issues outside of work’, which enabled the migrant nurses to continue their work in Japan.

“Even if I passed the Japanese national examination, my Japanese won’t be enough. [At my workplace] my Japanese colleagues know what level of Japanese I am at. So, they don’t make me feel overwhelmed, they understand what kind of Japanese language I am more likely to understand, so it’s easy to work here.” (More than 5 years.)

“The Japanese language is difficult because there are a lot of kanji. My preceptor always supports me when I can’t do something in my work. For example, the nursing summary at discharge is quite difficult. At first I did it by myself, and then they checked it. If I have any problems, they work with me.” (3–4 years.)

“I have a Japanese nurse friend who really helps me a lot. When I passed the Japanese national examination and brought my spouse to Japan, there were many procedures and many things I did not understand. My friend helped me then.” (1–2 years.)

3. Comfortable working environment

For this third category, the participants continued to work because they felt comfortable in a work environment with a ‘higher salary’ and ‘better working conditions’ than in their own country, ‘understanding of the characteristics of migrant nurses’ and ‘continued provision of Japanese language training’. In addition, the environment in which they could ‘help each other with EPA nurses in the same hospital’ supported their motivation to continue their work in Japan.

“Salaries are very good. For example, Japan is ten times higher than Indonesia.” (1–2 years.)

“If you experience working in healthcare in South East Asia, you’ll appreciate it in Japan. Work in my own country was busy, I didn’t get much rest, worked 16-h days and then I was on call. Sometimes I got a call right after I’ve just come back and it’s like, ‘We’re doing an emergency, please come in’.” (3–4 years.)

“There is also an EPA officer in the general affairs department, so I can ask for help them too. … The hospital where I’m working at now are caring about and understands EPA [nurses], so they know how much migrant nurses can work, so it’s easy to work here.” (3–4 years.)

“There are EPA care worker [at the same affiliated facility]. The hospital provides Japanese language training for them, but they said I should go in with them because they have a [Japanese] teacher here, so I can study there.” (More than 5 years.)

“It’s a bit difficult [to work in Japan if there is no connection with other migrant nurses]. When someone is in trouble, we help each other quite a lot. Like when we are studying, or when we are lonely or stressed out. We go somewhere together and talk. … Maybe that’s one of the reasons why I could have been working so hard in Japan.” (3–4 years.)

- Beneficial Nursing Experience in Japan

The participants felt that their nursing experience in Japan was beneficial to them and they continued in their work. This major factor consists of three categories: “high values of Japanese nursing practice”, “goals to achieve in Japan”, and “attachment to current job”.

1. High values of Japanese nursing practice

For this category, the participants evaluated Japanese nurses as having ‘sincere attitude’ for patients, ‘valuing care for patients’ daily life that was not implemented in their own country’, ‘providing patient-centred care’, and carrying out ‘efficient nursing work’. They also evaluated Japanese nurses’ ‘professional expertise’, which they recognized through ‘effective cooperation with other professions’ in the Japanese healthcare field.

“I prefer nursing in Japan (compared to my own country). I want to be a kind nurse. In Japan, they work carefully and diligently. They are neat and proper. They frequently check on the patient’s condition.” (1–2 years.)

“In Japan, nurses understand how the patients feel and what they need to do. They know exactly what they should do for patients. I think Japanese nurses are amazing.” (1–2 years.)

“In Japan, work is done in minutes and the daily workflow is well defined. In my country, there was a work schedule, but there was no set time. So, when one task was finished, they moved on to the next one, and so on; there was a waste of time because of the repetition of the same thing.” (More than 5 years.)

“Japanese nurses are able to assess the patient’s symptoms by themselves, consult with the doctor and then switch drugs. As soon as I consult with the patient and the doctor and they say it’s okay, I can do it.” (3–4 years.)

2. Goals to achieve in Japan

The participants had goals they wanted to achieve in Japan, such as ‘learning Japanese specialized nursing’, ‘supporting junior migrant nurses’, and ‘improve Japanese language skills’, which motivated them to continue their work in Japan.

“I want to study dialysis in order to become a certified nurse in Japan.” (More than 5 years.)

“My goal in Japan is to be able to teach EPA (nurses) like me to study together. Before I taught EPA (candidates) who hadn’t yet passed the Japanese national nursing examination so that they could pass. It’s very worthwhile.” (More than 5 years.)

“I want to be able to speak Japanese well and politely.” (1–2 years.)

3. Attachment to current job

The participants continued in their work because ‘they did not want to waste their efforts made so far’ in passing the Japanese national nursing examination, in their nursing experience in Japan, and in building human relationships in the workplace, and because they had adapted to their current working environment and felt that they have ‘no need to change their jobs’.

“[If I go to another country] I have to reset. I’ve been here [in Japan] for four years, and I thought I’d be wasting my Japanese nursing license and my job if I left Japan.” (3–4 years.)

“I’m comfortable now, so that’s good. No need to change.” (1–2 years.)

- Determination to live in Japan

Some participants came to Japan under the EPA with the intention of settling in Japan, and upon getting the Japanese nurse license, decided to live in Japan and continued their duties as a means of living in Japan. This major factor consists of two categories: “attractive life in Japan” and “migrated to Japan with family”.

1. Attractive life in Japan

The participants found life in Japan attractive because they could have a ‘safe daily life’ and ‘convenient living’ and they felt the ‘comfort of living with Japanese people’. ‘Interest in Japanese culture’ was also a motivation for continuing to live and work in Japan.

“Japan is a safe country. Japanese people are kind.” (More than 5 years.)

“Japan is convenient because it has everything we need for daily life.” (More than 5 years.)

“I have some Japanese friends. For example, if they told me they will pick me up at 10 am tomorrow, they actually come at exactly 10 am. I think that’s great.” (1–2 years.)

“I am interested in Japanese life and culture, so I want to continue (working in Japan), even though it is hard. I also want to travel.” (1–2 years.)

2. Migrated to Japan with family

For this category, the participants who moved to Japan with their families continued to work in Japan because ‘their families had adapted to Japanese life’ and ‘they want to raise their children in Japan’, where it is safe, there is sufficient medical care, good education, and less bullying.

“I have children in Japan now, so I can’t make the decision to go back (to my country) that easily. (My children) are already in school in Japan.” (More than five years.)

“I think it is safer to raise children in Japan. … I think there is less bullying compared to other countries. Also, I feel safer if my child goes to school by himself.” (3–4 years.)

## 4. Discussion

A web-based questionnaire survey and interviews were conducted to identify factors related to the job continuance of migrant nurses who came to Japan under the EPA and passed the national nursing examination. The factors related to their intention to quit their job from the results of the web-based questionnaire survey were human relationships in the workplace, the number of night shifts, a balance between work and private life, and job satisfaction with the work contents. The interview results showed that Generous Support from the Workplace, Beneficial Nursing Experience in Japan, and Determination to Live in Japan were related to the job continuance of migrant nurses. 

Although the sample size in this study was smaller compared to the survey report of 60 migrant nurses in 2013 [15], we believe it is sufficient since only data on a few migrant nurses were available in recent years [14].

### 4.1. Satisfaction with the Number of Night Shifts, Balance between Work and Private Life, and Contents of Work

The web-based questionnaire survey results showed that the higher the level of satisfaction regarding the number of night shifts, the balance between work and private life, and the content of work, the lower the intention to quit. However, all correlation coefficients that were significantly different were less than 0.5, indicating weak correlations. This is attributed to the small sample size.

The results of the web-based questionnaire survey are in line with previous studies that good working conditions encourage migrant nurses to work and migrate abroad [3,31]. Similar factors related to the Intention to Quit were reported in Saudi Arabia, which hosts a large number of migrant nurses [32]. It has also been reported that migrant nurses who came to Japan under the EPA appreciate the salaries in Japan [14]. In the interviews, migrant nurses also cited higher salaries and good working conditions in Japan than in their own countries as factors related to their job continuance. This was consistent with the social support, interpersonal relationships, and workload reported as factors that define job satisfaction among nurses in Asian cultures [33]. It can be inferred that the increase in job satisfaction among migrant nurses led to their job continuance.

As for the contents of work, the interview results showed that the migrant nurses valued Japanese nursing in terms of the sincere attitude of their supervisors and colleagues, the emphasis on care for patients’ daily life, patient-centred care, efficient nursing work, effective collaboration with other professions, and the nurses’ expertise. For these reasons, migrant nurses wanted to practice Japanese nursing and continued their work in Japan. Previous studies have indicated that migrant nurses from countries where the family provides daily living support to patients reported feeling strange with Japanese nursing, where nurses provide daily living support [14,34]. The participants in this study valued the nursing care of their supervisors and colleagues, who served as role models during the process of gaining nursing experience in Japan. This may have helped them to overcome their initial discomfort arising from nursing and cultural differences and to find value in Japanese nursing practice, which led them to continue their work in Japan. The interview results also indicated that the migrant nurses were satisfied with the contents of their work and continued because their supervisors made sure that the migrant nurses could be assigned to the department where they wanted to work. 

### 4.2. Support for Nursing Practice Required by Migrant Nurses

The interview results showed that even after passing the national nursing examination, migrant nurses need support in nursing tasks involving Japanese, such as communication and nursing records, and that receiving generous support from the workplace enabled them to continue their work. This confirms reports from previous studies that even if migrant nurses passed the national nursing examinations, they face difficulties in performing specialized nursing skills alone owing to insufficient Japanese language skills [17], as well as differences in working styles and nursing perspectives [14]. From the interview results, supervisors and colleagues at the workplace understood that it is difficult for migrant nurses to perform nursing work independently even after passing the national nursing examination. Accordingly, they checked the nursing records prepared by the migrant nurses and used easy-to-understand Japanese according to the migrant nurses’ Japanese language skills. In addition, some hospitals continued to provide study support to migrant nurses after they had passed the national nursing examination to help them learn the Japanese language. Since the role of nurses differs between the migrant nurse’s country and Japan [14], it is necessary to understand these differences and to support migrant nurses with guidance on Japanese nursing care.

### 4.3. Specific Support Needs of Migrant Nurses

#### 4.3.1. Problem Solving and Emotional Support in Private Life

The interview results revealed that migrant nurses living away from their home country need support from their Japanese supervisors and colleagues not only for problems related to nursing work but also for various issues in their private lives, as they had few family members and friends close by upon whom they could rely on. This is a different type of support needed from the Japanese staff, who draw a line between work and private life. The Japanese staff were willing to expand their roles to provide support beyond employment. The negative correlation in the web-based questionnaire survey between satisfaction with human relationships at work and intention to quit work was presumed to be related to the characteristics of migrant nurses, who find it difficult to solve problems not only in their work but also in their daily life in Japan if they do not have supportive relationships with their supervisors and colleagues. In addition, issues unique to migrant nurses, such as taking long vacations to temporarily return to their own country, also required understanding and support. 

The interview results indicate that an environment in which migrant nurses can provide mutual emotional support is a factor related to job continuance. As some previous studies reported that interaction between migrant nurses promotes the sharing of problems and stress relief [34], it is hoped that a mutual support system for migrant nurses beyond the hospitals will be established in the future.

#### 4.3.2. Migrated to Japan with Family

The interview results revealed that some migrant nurses felt that they wanted to raise their children in Japan, where it is safe and there is good medical care and education, and that they made the decision to migrate to Japan with their families when they had a foundation for life in Japan as a result of passing the Japanese national nursing examinations. Previous studies have reported that safety, education, and employment opportunities for families as well as migrant nurses are the advantages of migrating to developed countries [3] and that a stable life with their families in the destination country is a determining factor in their intention to stay in that country [35]. Getting married and living with one’s family is a high-priority issue for migrant nurses, as marriage and family care were the reasons why migrant nurses who came to Japan under the EPA left their jobs [10]. The interviews showed that one of the factors related to job continuance was that supervisors and colleagues supported the migrant nurses in the procedures to bring their families and even helped to find jobs for their families. If support measures for the immigration of migrant nurses who have passed the national nursing examinations and their families are established in the future, this may encourage migrant nurses to continue their work in Japan. 

#### 4.3.3. Professional Development Opportunities for Migrant Nurses

The interview results indicated that migrant nurses’ job satisfaction was linked to the support given to them by their supervisors and colleagues for tasks where they were deficient while trusting them according to their competence. It has been reported that restrictions on nurses’ work because they are migrant nurses leads to lower job satisfaction [36]; therefore, acknowledging the capacities of migrant nurses and encouraging them are imperative. Some migrant nurses have goals they want to achieve by working in Japan, such as learning Japanese and more specialized nursing. They also wanted to contribute in various opportunities such as providing support to junior migrant nurses. Kingma [5] reported that a workplace for personal growth and professional development improves job satisfaction and retention among migrant nurses. A future challenge for Japan is how to build on the careers of migrant nurses [37]. To respond to this challenge, it is desirable to build opportunities for personal growth and professional development that can utilize migrant nurses’ strengths and experiences from their own countries, rather than treating them as nurses who cannot speak Japanese.

### 4.4. Risk and Prevention of Burnout among Migrant Nurses 

The work environment, such as human relationships and work–life balance, which were related to nurses’ intention to quit their job in this study, are similar to burnout factors that cause turnover [18,19,38]. From previous research with Japanese nurses, the perception of a heavy workload affected burnout [38]. The perceived work burden of migrant nurses working in Japan is high [16] and they are at high risk of burnout as they have problems communicating in an unfamiliar language [21]. From the interview results, it is considered that the Japanese nurses in the workplace understood the migrant nurses’ Japanese language level and provided generous support for their nursing tasks, thereby preventing the migrant nurses from burnout due to high workload. 

The study of Japanese nurses reported that satisfaction with nursing care was negatively associated with burnout [38]. The results of this study also showed that satisfaction with the work content was negatively associated with migrant nurses’ intention to quit. This is consistent with the findings of a previous study showing that job satisfaction of migrant nurses is negatively associated with burnout [39]. The unmet expectations of migrant nurses in their new country may have a negative impact on their integration and retention in their new workplace [21]. Migrant nurses working in Japan experience differences from nursing practice in their own country [14] and difficulties that limit the tasks they can perform independently compared to Japanese nurses [17]. The gap between expected work and reality can cause burnout in migrant nurses. The interview results showed that the migrant nurses found value in Japanese nursing practice, which led to their continued employment. Supervisors also trusted and applauded the migrant nurses’ work, and supported them to achieve their preferred working style. This support from Japanese nurses was considered to have reduced the risk of burnout by respecting the migrant nurses’ expertise and requests.

### 4.5. Implications from the Asian Scope for Countries Receiving Migrant Nurses

In Asia, the major host countries for migrant nurses are Singapore, Malaysia, and Japan [7]. However, considering the rapid ageing of the population in Asian countries, more countries may invite migrant nurses in the future to provide much-needed care for an older population. Even though Asian countries share a general Asian culture, each country has its own language, specific culture, and nursing education, giving rise to the difficulties migrant nurses often experience in the host countries. In Singapore, migrant nurses account for approximately 20% of the workforce [7] and they experience difficulties owing to differences in nursing education and nursing roles, limited opportunities for promotion, and inequalities with Singaporean employees [40]. It is easy to imagine that in Asian countries where few people speak English such as Japan, Thailand, and Korea, migrant nurses will have additional difficulties working [7]. The most common difficulty faced by migrant nurses working in Japan was reportedly speaking and understanding the Japanese language [14], which limits their opportunities for independent nursing practice [17]. It is challenging to overcome language barriers in Japan where the languages are very original and use their own alphabet. It will be a mutual challenge in Asian countries to support migrant nurses, who face barriers as foreigners in addition to the hard workload of the nursing profession, to work safely and longer in their destination countries. This research explored the possible support for migrant nurses for them to continue their jobs in their host countries. Migrant nurses have different needs, such as continuous language learning and long vacations to return to their home. In accepting migrant nurses, it is necessary to disseminate this information to each facility. In addition, opportunities for host nurses to understand the characteristics and unfamiliar tasks of migrant nurses are also desirable in order to support migrant nurses’ employment.

### 4.6. Limitations and Implications for Future Study

The facilities requested for research cooperation included some hospitals where the target migrant nurses had already left Japan. Because of this, the response rate for the web-based questionnaire survey was low, limiting the generalizability of the study results. 

The scale psychometrics were not performed; therefore, the results should be cautiously interpreted. As this study anticipated a small sample size and limited data collection for factors related to job continuance, it would be desirable to conduct a quantitative survey of a larger sample of migrant nurses in the future, including more factors that may be related to job continuity, such as level of Japanese language skills, desire to migrate to Japan, and family immigration status, which were not surveyed in this study. A large sample size also allows the analysis to search for factors that are strongly associated with turnover.

As the interviews were conducted in Japanese, there were limits to what the study participants could talk about in terms of their own experiences and thoughts, especially for those who had been in Japan for a short period of time, and there might be cases where their true feelings and detailed nuances were not fully captured. In the future, a survey of nurses who returned to their home countries without continuing their work in Japan would allow for a deeper exploration of the factors related to job continuance among migrant nurses. As a cultural aspect, consideration of the religion of migrant nurses is also necessary, but these data could not be collected in this study.

In addition, as the interview results were analysed based on the subjective views of the migrant nurses, in the future it is also necessary to examine the factors related to job continuance from a comprehensive perspective, including the narratives of the Japanese staff. Furthermore, the results of this study indicated that supervisors and colleagues at workplaces provide extensive professional and private support to migrant nurses; it was predicted that this would place a heavy burden on the workplaces. Therefore, it is also desirable to investigate the support needs of supervisors and colleagues who work with migrant nurses. Despite these limitations, the results of the study were consistent with other studies of migrant nurses and highlighted the importance of infusing evidence-based supportive policies and practices into the host hospitals.

## 5. Conclusions

This study found that a supportive work environment, generous support from supervisors and colleagues, high values for Japanese nursing practice, and a desire to migrate to Japan were factors related to job continuance for migrant nurses who came to Japan under the EPA and passed the national nursing examination. Understanding the characteristics of migrant nurses, including opportunities to return home, language learning, support for unfamiliar nursing tasks, and providing generous support will enable migrant nurses to continue working in Japan. The results of this study will be useful in other countries that accept migrant nurses from Indonesia, the Philippines, and Vietnam to establish support measures for their employment. In the future, it is necessary to conduct surveys of Japanese nurses working with migrant nurses and establish a system to ensure that migrant nurses receive appropriate support.

## Figures and Tables

**Table 1:** Characteristics of participants responding to the web-based questionnaire survey.

		*n* = 40	
Attribute		*n*	%
Gender identification	Male	11	27.5
	Female	29	72.5
Country of origin	Indonesia	16	40.0
	Philippines	14	35.0
	Vietnam	10	25.0
Marital status	Married	21	52.5
	Unmarried	19	47.5
Presence of children	Presence	17	42.5
	None	23	57.5
Number of visits to Japan as a nurse	First time	36	90.0
	Second time	4	10.0
	Mean (+/− SD)	Range (year)	
Age	32.3 (4.2)	25–41	
Length of stay in Japan (year) *n* = 30 ^1^	5.6 (3.6)	1–12	
Years of experiences as a nurse in Japan (year)	3.9 (3.1)	0.6–10	
Years of experiences as a nurse in their own country (year)	3.7 (1.6)	2–9	

SD, standard deviation. ^1^ The survey was started when the questions were not yet reflected on the web. This resulted in missing responses for the first 10 respondents.

**Table 2:** Relationship between Intention to Quit by basic attributes, and Current Job Satisfaction.

			*n* = 40
		Mean (+/− SD)	Range (Min–Max)
Total score of Intention to Quit		7.9 (3.3)	3–12
Basic attributes		Mean (+/− SD)	*p*-value
Gender identification	Male	7.4 (3.6)	0.633
	Female	8.1 (3.3)	
Country of origin	Indonesia	7.7 (3.5)	0.416
	Philippines	8.7 (3.2)	
	Vietnam	7.0 (3.4)	
Marital status	Married	7.6 (3.4)	0.611
	Unmarried	8.2 (3.3)	
Presence of children	Presence	8.9 (3.4)	0.095
	None	7.1 (3.2)	
Number of visits to Japan	First time	7.8 (3.4)	0.499
	Second time	9.0 (2.9)	
		Correlation coefficient	*p*-value
Age		0.000	0.999
Length of stay in Japan *n =* 30 ^2^		−0.004	0.982
Years of experiences as a nurse in Japan		−0.139	0.392
Years of experiences as a nurse in their own country		−0.029	0.858
Current Job Satisfaction		Correlation coefficient	*p*-value
Salary		−0.189	0.242
Content of work		−0.453	0.003 **
Support from supervisor		−0.309	0.053
Relationships in workplace		−0.353	0.026 *
Training in workplace		−0.202	0.212
Number of night shifts *n =* 39 ^3^		−0.394	0.013 *
Balance between work and private life		−0.437	0.005 **

* *p* < 0.05, ** *p* < 0.01, SD: standard deviation. ^2^ The survey was started when the questions were not yet reflected on the web. This resulted in missing responses for the first 10 respondents. ^3^ One case where two options were answered simultaneously was treated as a missing value.

**Table 3:** Characteristics of interview participants.

	County of Origin	Age	Gender	Marital Status	Years of Experiences as a Nurse in Japan
A	Indonesia	30 s	Woman	Married	>5 years
B	Indonesia	30 s	Man	Married	>5 years
C	Indonesia	30 s	Man	Married	1–2 years
D	Philippines	30 s	Woman	Married	>5 years
E	Philippines	30 s	Man	Married	3–4 years
F	Philippines	30 s	Man	Unmarried	3–4 years
G	Vietnam	20 s	Woman	Married	1–2 years
H	Vietnam	20 s	Woman	Unmarried	1–2 years
I	Vietnam	20 s	Woman	Unmarried	1–2 years

**Table 4:** Factors related to job continuance of migrant nurses.

Major Category	Category	Subcategory
Generous support from the workplace (14)	Dependable supervisor (5)	Easy to ask for help
		Trusted and entrusted migrant nurse’s work
		Applauds their work
		Resolving difficulties in work and private life
		Enabling them to achieve their preferred working style
	Good relationships with colleagues (4)	Understand the capability of migrant nurses
		Kind teaching
		Support migrant nurses with unfamiliar tasks
		Help with issues outside of work
	Comfortable working environment (5)	Higher salary than in their own country
		Better working conditions than in their own country
		Understanding of the characteristics of migrant nurses
		Continued provision of Japanese language training
		Help each other with EPA nurses in the same hospital
Beneficial nursing experience in Japan (11)	High values of Japanese nursing practice (6)	Sincere attitude of Japanese nurses
		Nursing that values care for patients’ daily life that was not implemented in their own country
		Providing patient-centred care
		Efficient nursing work
		Effective cooperation with other professions
		Nurses’ expertise recognized
	Goals to achieve in Japan (3)	Learn Japanese specialized nursing
		Support junior migrant nurses
		Improve Japanese language skill
	Attachment to current job (2)	A desire not to waste the efforts made so far
		No need to change jobs
Determination to live in Japan (6)	Attractive life in Japan (4)	Safe daily life
		Convenient living
		Comfort of living with Japanese people
		Interest in Japanese culture
	Migrated to Japan with family (2)	Families adapting to Japanese life
		Desire to raise children in Japan

## Data Availability

The data collected in this study are not available to the public for reasons of informed consent and confidentiality but may be obtained from the corresponding author upon reasonable request.

## Public Involvement Statement

No public involvement in any aspect of this research.

## Guidelines and Standards Statement

This manuscript was drafted against the Strengthening the Reporting of Observational Studies in Epidemiology (STROBE) guidelines for cross-sectional studies.

## References

[1] World Health Organization (2020). Nursing and Midwifery. https://www.who.int/newsroom/fact-sheets/detail/nursing-and-midwifery.

[2] Kline D.S. (2003). Push and pull factors in international nurse migration. J. Nurs. Scholar..

[3] Kingma M. (2001). Nursing migration: Global treasure hunt or disaster-in-the-making?. Nurs. Inq..

[4] World Health Organization State of the World’s Nursing 2020: Investing in Education, Jobs and Leadership. https://www.who.int/publications/i/item/9789240003279.

[5] Kingma M. (2008). Nurses on the move: Diversity and the work environment. Contemp. Nurse..

[6] World Health Organization International Nurse Mobility: Trends and Policy Implications. No. WHO/EIP/OSD/2003.3. https://apps.who.int/iris/handle/10665/68061.

[7] Matsuno A. (2009). Nurse Migration: The Asian Perspective. ILO/EU Asian Programme on the Governance of Labour Migration. https://www.ilo.org/asia/publications/WCMS_160629/lang--en/index.htm.

[8] JICWELTS Acceptance Edition for 2020: Brochure on the Acceptance of Foreign Nurse and Care Worker Candidates under the EPA. https://jicwels.or.jp/files/EPA_2020_pamph.pdf.

[9] Ministry of Health, Labour and Welfare (2019). Overview of Acceptance of Foreign NURSE and care Worker Candidates under the Economic Partnership Agreement (EPA). https://www.mhlw.go.jp/content/000639886.pdf.

[10] Hirano Y. (2019). Reception of Foreign Nurses and Japan: Focusing on Indonesian Nurses’ Return and CAREER development. New Med. World Wkly..

[11] Japanese Nursing Association (2023). News Release: Results of the 2022 Hospital Nursing Survey. https://www.nurse.or.jp/home/assets/20230301_nl04.pdf.

[12] Ministry of Health, Labour and Welfare (2022). Publication of the Number of Successful Candidates for the 111th National Nursing Examination for Foreign Nurses under the Economic Partnership Agreement (EPA) and the NAMES of their Institutions. Attachment 2. https://www.mhlw.go.jp/stf/newpage_24807.html.

[13] Furukawa E., Seto K., Matsumoto K., Hasegawa T. (2012). A questionnaire survey of Economic Partnership Agreement (EPA) host facilities for foreign nurses. JHM.

[14] Efendi F., Chen C.M., Nursalam N., Indarwati R., Ulfiana E. (2016). Lived experience of Indonesian nurses in Japan: A phenomenological study. Jpn. J. Nurs. Sci..

[15] Japan International Corporation of Welfare Services (JICWELS) (2013). Report of the Ministry of Health, Labour and Welfare’s special Measures for Securing Nursing Personnel: Survey Project Report on EPA Nurses. https://jicwels.or.jp/files/E69CACE69687.

[16] Yamamoto T., Mizukami K. (2018). Occupational Stress among Nurses Working in Japan through the Economic Partnership Agreement (EPA). Off. J. Jpn. Prim. Care Assoc..

[17] Ino K., Watanabe Y. (2014). The Practice of Nursing Techniques by Foreign Nurses Who Came to Japan under an Economic Partnership Agreement Program. Kango Gijutsu.

[18] Kelly L.A., Gee P.M., Butler R.J. (2021). Impact of nurse burnout on organizational and position turnover. Nurs. Outlook.

[19] Shah M.K., Gandrakota N., Cimiotti J.P., Ghose N., Moore M., Ali M.K. (2021). Prevalence of and Factors Associated With Nurse Burnout in the US. JAMA Netw. Open.

[20] Maslach C., Jackson S.E. (1984). Burnout in organizational settings. Appl. Soc. Psychol. Annu..

[21] Roth C., Berger S., Krug K., Mahler C., Wensing M. (2021). Internationally trained nurses and host nurses’ perceptions of safety culture, work-life-balance, burnout, and job demand during workplace integration: A cross-sectional study. BMC Nurs..

[22] Hirano O.Y., Ogawa R., Ohno S. (2012). A comparative study of Filipino and Indonesian candidates for registered nurse and certified care worker coming to Japan under Economic Partnership Agreements: An analysis of the results of questionnaire surveys on the socioeconomic attribution of the respondents and their motivation to work in Japan. Jpn. J. Southeast Asian Stud..

[23] Aijo R., Katayama M., Kitaoka K. (2017). Relationship between job satisfaction and intention to remain among nurses working in a hospital in Japan. J. Wellness Health Care.

[24] Kato E., Ozaki F. (2011). Study of related factors among job-continuing intention, job satisfaction and burnout for nurses in mid-career. J. Jpn. Acad. Nurs. Adm. Policies.

[25] Arakawa C. (2011). Factors affecting the resignation of female nurses: Longitudinal study of nurses working at hospitals with more than 200 beds in the general ward in the Kanto Region. J. Int. Nurs. Res..

[26] Watanabe R., Arakida M., Suzuki S. (2010). Individual and organizational factors influencing turnover among young nurses: Comparison between one and five years of experience. Jpn. J. Nurs. Sci..

[27] Kimura Y. (2014). Legitimate peripheral participation in EPA nurses: What supports continuity of ward work. Obirin Gengo Kyouiku Ronsou..

[28] Doyle L., McCabe C., Keogh B., Brady A., McCann M. (2020). An overview of the qualitative descriptive design within nursing research. J. Res. Nurs..

[29] Furuya H., Tani F. (2008). The burnout process model from the occurrence through intention to quit among nurses. Jpn. J. Nurs. Sci..

[30] Gregg M., Asahara K., Yokoyama Y. (2016). Understanding How to Conduct and Summarise Qualitative Research: Towards Becoming an Expert in Nursing Research.

[31] Li H., Nie W., Li J. (2014). The benefits and caveats of international nurse migration. Int. J. Nurs. Sci..

[32] Alreshidi N.M., Alrashidi L.M., Alanazi A.N., Alshammeri E.H. (2021). Turnover among foreign nurses in Saudi Arabia. J. Public Health Res..

[33] Sriratanaprapat J., Songwathana P. (2013). Nurses’ Job Satisfaction within the Context of Asian Cultures: A Concept Analysis. Pac. Rim Int. J. Nurs. Res..

[34] Yamamoto S., Higuchi M. (2015). Experiences of foreign nurse candidates in Japan under the Economic Partnership Agreement. J. Int. Health.

[35] Humphries N., Brugha R., McGee H. (2009). “I won’t be staying here for long”: A qualitative study on the retention of migrant nurses in Ireland. Hum. Resour. Health.

[36] Adhikari R., Melia K.M. (2015). The (mis) management of migrant nurses in the UK: A sociological study. J. Nurs. Manag..

[37] Hirano Y., Hirano Y., Yoneno M. (2021). Economic Partnership Agreements and the international migration of nurses ‘double standard employment’. Foreign Nurses Working in Japan: Assessments of the EPA Program.

[38] Yokoo Y., Suzuki E., Hiramoto Y., Ujiie Y. (2022). Factors Associated with Burnout Among Female Nurses Working in Specialty Hospitals. Jpn. Health Med. Assoc..

[39] Batayneh M.H., Ali S., Nashwan A.J. (2019). The burnout among multinational nurses in Saudi Arabia. Open J. Nurs..

[40] Choi S., Lenore L. (2012). Gender, citizenship, and women’s ‘unskilled’ labour: The experience of Filipino migrant nurses in Singapore. Can. J. Women Law.

